# Nutritional Adequacy and Health Outcomes

**DOI:** 10.3390/nu17152465

**Published:** 2025-07-29

**Authors:** Enbo Ma

**Affiliations:** 1 Health Promotion Centre, Fukushima Medical University, Fukushima City 960-1295, Japan; mae@fmu.ac.jp; 2 Department of Epidemiology, School of Medicine, Fukushima Medical University, Fukushima City 960-1295, Japan

Adequate intake refers to the daily amount of a nutrient sufficient to meet the needs of most individuals in a population group. When the estimated average requirement for a nutrient is not available, adequate intake is established as a formal reference value based on expert judgment, informed by the latest research [1]. Notably, evaluating nutrition further in relation to health status requires more than a single nutrient or food [2]. Food intakes, dietary patterns, and behaviors must also be considered to maintain good health and prevent or treat diseases. In nutrition practice, routinely monitoring an individual’s nutritional status and improving their nutrition knowledge and healthy dietary behaviors is essential. This Special Issue served this purpose by publishing two reviews and four original research articles.

Glutamine is a conditionally essential amino acid for maintaining metabolic functions [3]. Wunderle et al. investigated the impact of glutamine in a randomized clinical trial among 234 patients in Swiss hospitals and found that low plasma levels of glutamate were independently associated with 30-day mortality, suggesting glutamate is an independent prognostic factor among inpatients at nutritional risk.

Iron deficiency was a common nutritional problem in China [4]. Optimizing nutrient availability within the food system can more effectively address nutritional deficits than solely increasing agricultural output [5]. In another study on a local self-supply system in China, Zhao et al. found that 12 out of 31 provinces faced challenges in meeting iron intake requirements, with deficiencies ranging from 11% to 108%. However, 52% (folate) to 90% (vitamin B12) of agricultural output was available for dietary provisioning nationwide, highlighting the need to address micronutrient deficiency risk in global food trade to ensure a secure and equitable nutrition supply.

Le Turc et al. performed a survey of 639 Portuguese and French students, explored consumer knowledge of the dietary importance of fruits and vegetables, and revealed that most participants were young females (68.9%) with a good education (76%) and average body weight, with 99.1% women and 96.4% men falling into the overestimation of their dietary behavior. This highlights the need for targeted educational programs to enhance nutrition literacy, particularly among less informed demographic groups. Additionally, Lopes et al. found in a scoping review that interventions using native foods—recognizing their nutrient-rich properties [6]—show promising results in enhancing health, nutritional outcomes, cultural identity, and food security, demonstrating their potential for broader public health applications and the value of participatory approaches for sustainable interventions.

This Special Issue also included two articles on dietary patterns. In a cross-sectional survey assessing the Mediterranean Diet Serving Score among 139 Spanish university students, Béjar found that an overestimation of dietary behavior may be associated with lower adherence to the Mediterranean diet, which is linked to multiple health benefits and sustainability [7]. Furthermore, Aliberti et al. in a study on global longevity patterns identified common factors in longevity blue zones, including hilly altitudes between 355 and 600 m, mild climates with temperatures between 17.4 and 23.5 °C, and predominantly Mediterranean or plant-based diets. These factors help protect populations from non-communicable chronic diseases and slow the aging process.

In summary, this Special Issue focuses on studying adequate nutrient intake, food consumption knowledge, and adherence to healthy dietary patterns among various populations globally, providing novel findings and recommendations for healthy nutrition practices.
